# An urban crowd flow model integrating geographic characteristics

**DOI:** 10.1038/s41598-023-29000-5

**Published:** 2023-01-30

**Authors:** Yu Zhang, Sheng Wu, Zhiyuan Zhao, Xiping Yang, Zhixiang Fang

**Affiliations:** 1 Academy of Digital China (Fujian), Fuzhou University, Fuzhou, China; 2 State Key Laboratory of Information Engineering in Surveying, Mapping and Remote Sensing, Wuhan University, Wuhan, China; 3 Key Laboratory of Spatial Data Mining &Information Sharing of Ministry of Education, Fuzhou, China; 4 Ministry of Education Fujian Collaborative Innovation Center for Big Data Applications in Governments, Fuzhou, China; 5 School of Geography and Tourism, Shaanxi Normal University, Xi’an, China; 6 The Digital Economy Alliance of Fujian, Fuzhou, China; 7 Key Laboratory of Urban Land Resources Monitoring and Simulation, Ministry of Natural Resources, Shenzhen, China

**Keywords:** Information theory and computation, Socioeconomic scenarios, Sustainability, Mathematics and computing

## Abstract

Predicting urban crowd flow spatial distributions plays a critical role in optimizing urban public safety and traffic congestion management. The spatial dependency between regions and the temporal dynamics of the local crowd flow are two important features in urban crowd flow prediction. However, few studies considered geographic characteristic in terms of spatial features. To fill this gap, we propose an urban crowd flow prediction model integrating geographic characteristics (FPM-geo). First, three geographic characteristics, proximity, functional similarity, and road network connectivity, are fused by a residual multigraph convolution network to model the spatial dependency relationship. Then, a long short-term memory network is applied as a framework to integrate both the temporal dynamic patterns of local crowd flow and the spatial dependency between regions. A 4-day mobile phone dataset validates the effectiveness of the proposed method by comparing it with several widely used approaches. The result shows that the root mean square error decreases by 15.37% compared with those of the typical models with the prediction interval at the 15-min level. The prediction error increases with the crowd flow size in a local area. Moreover, the error reaches the top of the morning peak and the evening peak and slopes down to the bottom at night.

## Introduction

Urban crowd flow dynamics are the fundamental data used in many smart city applications, such as urban traffic congestion management, public facility planning, epidemic prevention and control^1,2^. However, due to the complex nature of human dynamics caused by various spatial and temporal constraints and approaches to overcome these constraints, predicting urban crowd flow is challenging. The current crowd flow in a certain area is jointly affected by the past local crowd flow and the crowd flow in the surrounding areas. Both the temporal dynamic patterns of the local area and the spatial dependency relationships between the local area and the related regions need to be considered for a well-designed urban crowd flow prediction model. Therefore, existing prediction models can be divided into two types: temporal feature-based models and spatial feature-based models.

In terms of the temporal feature-based model, urban crowd flow distribution prediction is modeled as a temporal forecasting problem, and classic time series models are first applied^3^. The development of machine learning, such as long short-term memory (LSTM), has further improved the accuracy and ability to predict crowd flow based on time relationships^4^. In terms of the spatial feature-based model, modeling and integrating the complex and dynamic spatial relationships between the local areas and the related areas challenges traditional methods. The emergence of deep learning models, such as convolutional neural networks (CNNs)^5^, graph convolutional networks (GCNs)^6^ and residual multigraph convolutional networks (RMGCNs)^7^, has promoted spatial relation modeling and integration. However, in these studies, only some simple relations between adjacent regions (e.g., connectivity relationships) were considered, and many meaningful but complex spatial features have not been well-integrated.

Geological characteristics, such as regional function characteristics and connectivity strength, play important roles in predicting crowd flow. Similar regional functions between areas imply similar crowd flow dynamics rhythm. For example, the crowd flow in a residential area exhibits substantial loss in the morning and recovers at night. As a result, local crowd flow dynamic patterns can contribute to improving the prediction performance for crowd flow in areas with similar regional functions. Moreover, connectivity strength can effectively measure the impacts from both adjacent regions and distant areas. For example, urban expressways can considerably improve the connectivity strength between distant regions by reducing the travel time. Therefore, only considering the adjacent areas to model the spatial relationships may ignore the impacts of crowd flow from distant but highly connected areas. A method that can integrate multiple spatiotemporal relationships is needed to improve the urban crowd flow prediction performance.

In this study, we aim to answer the following two questions: (1) How do geographic characteristics affect the prediction of urban crowd flow? (2) What is the performance of the proposed method compared to the method without considering geographic characteristics (e.g., LSTM, RMGCN) and how are the prediction errors distributed on the spatial and temporal dimensions?

This paper is organized as follows: In the related work section, a comprehensive overview is given. Then, the methodology of this paper is introduced. Next, the experiments and results, the experimental data and result analysis are presented. We discuss related results and draw several conclusions in the final section.

## Related work

According to the parametric requirements, existing urban crowd flow prediction models can be divided into two types: parametric and nonparametric. The parametric model is built based on a regression function, and the unknown parameters need to be estimated based on the benchmark dataset before predicting the regional crowd flow. For example, Pappalardo et al. proposed an analytical framework to nowcast the population count, well-being, and economic development based on mobile phone data^8^. Classic models, such as the historical average model^9^, autoregressive integrated moving average model (ARIMA)^10^ and Kalman filter^11^ all belong to this type. The historical average model uses the average value of traffic over historical moments as the predicted value. The ARIMA model proposed by Box and Jenkins^12^ regards the crowd flow dynamics in a local area as a time series and predicts the crowd flow by curve fitting and parameter estimation based on historical data. The Kalman filter establishes a linear system state equation and uses the crowd flow at the previous moment to obtain a prediction. However, traditional parametric models are not adept at addressing features of complexity and uncertainty (e.g., nonlinear patterns between variables) in urban crowd flow prediction problems.

The nonparametric model requires few basic assumptions between available variables and outperforms traditional parametric models in addressing complex features. The relationships between the dependent variables and the independent variables are learned and derived from historical data to establish an approximate model^13^. Typical approaches include K-nearest neighbors (KNN)^14^, Bayes^15^, support vector regression (SVR)^16^ and deep learning^17,18^, which have been widely used in urban crowd flow prediction applications as well as many other related fields. Recently, the deep learning model has developed rapidly due to its outstanding performance in integrating temporal and spatial characteristics of crowd flow dynamics^19^.

Integrating different spatial and temporal features with different matched deep learning models results in a varying performance. Early neural network models only considered temporal characteristics^20,21^. For example, Fu et al. used an LSTM model and a gated recurrent unit model to predict traffic flow^22^. Tian et al. also used an LSTM model to predict traffic speed and traffic flow. To better integrate spatial features, many deep learning models have been widely investigated and applied^23–25^. Chen et al. used an artificial neural network to predict the population in each grid from a neighborhood perspective^26^. Guangyuan et al. used a convolution LSTM model to predict the spatiotemporal distribution of mobile phone users at a fine-grained temporal resolution^27^. Zhang et al. proposed a spatial and temporal residential network model to predict crowd flows^7^. Wu et al. combined a CNN and LSTM to model the spatial and temporal relationships between regions, respectively, to construct a prediction model^28^. In terms of the spatial relationships, GCNs exhibit better modeling performance^29^. For example, Chai et al. used a GCN model to construct various spatial relationships between stations to predict bicycle traffic^30^. Sun et al. proposed a multi-view GCN (MVGCN) model to construct spatial correlations and interactions between irregular regions to predict crowd flow^31^.

However, many meaningful geographical features are rarely considered in spatial features during the above studies. The prediction errors and the effectiveness of different features lack insightful investigation and comprehension.

## Methodology

### Problem definition

We divide the study area into regular grids of equal size, and each grid is represented by $v_{i} ;\,{\varvec{V}} = \{ v_{1} ,v_{2} , \ldots ,v_{N}$} is the set of all the grids, and *N* represents the total number of grids, where $i \in \left[ {1,N} \right]$. A graph ***G*** = (***V***, ***A***) is constructed to represent the spatial relationships between grids. Each grid is regarded as a node, and the spatial link between each pair of grids is regarded as an edge. ${\varvec{A}} \in {\varvec{R}}^{N \times N}$ represents the spatial relation matrix between grids. ${\varvec{X}}_{t} \in {\varvec{R}}^{N}$ represents the crowd flow for all grids at time *t*. Predicting urban crowd flow can be modeled by constructing the function *F*(·) to calculate ***X***_*t*_ based on the spatial relation matrix ***A*** and the historical urban crowd flow ${\varvec{X}}_{t - 1} ,{\varvec{X}}_{t - 2} ,{\varvec{X}}_{t - H + 1}$ in a time window *H* (Eq. 1).

1 $${\varvec{X}}_{t} = F\left( {{\varvec{A}}; \left( {{\varvec{X}}_{t - 1} ,{\varvec{X}}_{t - 2} ,{\varvec{X}}_{t - H + 1} } \right)} \right)$$

### The framework of the proposed method

As shown in Supplementary Fig. 1, we first use mobile phone data to extract the crowd flow in each grid at every moment. Then, we propose three geographic characteristics to measure the spatial dependence from different perspectives and adopt the RMGCN to model these three features. Finally, an LSTM model is applied to integrate dependence relationships on both the temporal and spatial dimensions.

### Graph construction

Three graphs were constructed based on geographic characteristics from different perspectives (Supplementary Fig. 2): proximity, functional similarity, and connectivity relationships.

The proximity is measured by the adjacent relation. The proximity characteristic is derived from Tobler’s first law (TFL), which states that “everything is related to everything else, but near things are more related than distant things”^32^. We believe that the crowd flow in a grid could be strongly affected by nearby grids. For intuitive purposes, we adopt the Moore neighborhood (i.e., the eight grids that surround the central grid) to measure the proximity relationship (Eq. 2).

2 $${\varvec{A}}_{ij}^{J} = \left\{ {\begin{array}{*{20}l} {1,} \hfill & {\quad if\,v^{j} \,belongs\,to\,the\,8\,grids\,that\,surround\,v^{i} } \hfill \\ {0,} \hfill & {\quad {\text{else}}} \hfill \\ \end{array} } \right.$$

where ${\varvec{A}}_{ij}^{J}$ represents the proximity between *v*^*i*^ and *v*^*j*^.

The functional similarity is measured based on the similarity of the geographical properties. The functional similarity characteristic is derived from the “Third Law of Geography”, which states that “the more similar geographic configurations of two points (areas), the more similar the values (processes) of the target variable at these two points (areas)”^33^. According to this law, areas with similar context will have similar features. As a result, the crowd flow tends to be similar for grids with similar urban functions. For example, the crowd flow in areas with middle schools or office buildings share similar crowd flow patterns. Considering the accessibility of the data, we adopt the shared structure of different point of interest (POI) categories to represent the local geographical property. A simple method is constructed based on the POI shared structure to measure the functional similarity. In terms of the calculation method, we regard each grid as a vertex and measure the similarities of the functions between the grids (Eq. 3).

3 $${\varvec{A}}_{ij}^{P} = sim\left( {{\varvec{poi}}^{{\varvec{i}}} ,{\varvec{poi}}^{{\varvec{j}}} } \right)$$

The connectivity is measured by the shortest path distances between regions based on the road network. The connectivity characteristic is derived from the combination of the “Second Law of Geography” and the TFL. The “Second Law of Geography” implies the spatial heterogeneity of geographical phenomena and states that “geographic variables exhibit uncontrolled variance”^34^. According to this law, the effectiveness of a spatial model integrating geographical features relies on the location of the analysis areas, especially distant areas. However, the connectivity characteristics (e.g., express road in two cities) mean that distant areas can affect each other directly, making the TFL valid again. A shorter distance indicates a stronger connectivity relation. To improve the calculation efficiency, we regard two grids as unconnected if their shortest path distance exceeds threshold *δ*. Threshold *δ* can be set by referring to the prediction time step. In addition, considering that the grids surrounding a local grid have been modeled by the proximity relationship, these grids are not considered. The calculation equation is as follows:

4 $${\varvec{A}}_{i,j}^{C} = \left\{ {\begin{array}{*{20}l} {\frac{1}{{d\left( {v^{i} ,v^{j} } \right)}},\quad } \hfill & {0 < d\left( {v_{i} ,v_{j} } \right)} \hfill & { < \delta \quad and\quad {\varvec A}_{{i,j}}^{J} = 0} \hfill \\ {0,} \hfill & {esle} \hfill & {} \hfill \\ \end{array} } \right.$$

5 $$d\left( {v^{i} ,v^{j} } \right) = min\left( {dist\left( {v^{i} ,v^{j} } \right)} \right)$$

where $dist\left( {v^{i} ,v^{j} } \right)$ represents the Euclidean distance between *v*^*i*^ and *v*^*j*^, and $d\left( {v^{i} ,v^{j} } \right)$ is the shortest distance, *min* is the minimum function, and ${\varvec{A}}_{i,j}^{C}$ represents the connectivity strength.

### Graph fusion

To better model the geographic characteristics between grids, we need to perform graph fusion and merge graphs composed of different types of spatial relations into one graph. We perform graph fusion using weighted summation. We first normalize the three spatial relation matrices to handle the large value differences between different graphs (Eq. 6).

6 $${\varvec{L}} = {\varvec{I}} - {\varvec{D}}^{1/2} {\varvec{AD}}^{1/2}$$

7 $$\varvec{DD} = \left[ \begin{array}{cccc} \mathop \sum \limits_{{j = 0}}^{{n - 1}} \varvec{A}_{{0,j}} &\quad 0&\quad \cdots &\quad 0 \\ 0&\quad\mathop \sum \limits_{{j = 0}}^{{n - 1}} \varvec{A}_{{1,j}}&\quad\cdots &\quad0 \\ \vdots &\quad\vdots &\quad\vdots &\quad\vdots \\ 0&\quad0&\quad\cdots&\quad\mathop \sum \limits_{{j = 0}}^{{n - 1}} \varvec{A}_{{n - 1,j}} \\ \end{array} \right]$$

where ***A*** represents the spatial relation matrix (${\varvec{A}} \in \left[ {{\varvec{A}}^{J} ,{\varvec{A}}^{P} ,{\varvec{A}}^{C} } \right]$). ***D*** represents the corresponding degree matrix, and the calculation method is shown in Eq. (7). ***L*** represents the normalized spatial relation matrix. ***I*** is the identity matrix.

We normalize the fused spatial relation matrices according to Eq. (8) to obtain the normalized proximity matrix ***L***^*J*^, functional similarity matrix ***L***^*P*^, and road network connectivity matrix ***L***^*C*^. Then, the weighted summation of these three normalized spatial relation matrices is calculated.

8 $${\varvec{L}}^{Fu} = {\varvec{W}}^{0} \odot {\varvec{L}}^{J} + {\varvec{W}}^{1} \odot {\varvec{L}}^{P} + {\varvec{W}}^{2} \odot {\varvec{L}}^{C}$$

where ***L***^*Fu*^ is the spatial relation matrix after weighted summation. ***W***^0^, ***W***^1^, and ***W***^2^ are trainable parameters.

### Residual graph convolution

To better capture the geographic characteristics between regions, we use a residual GCN for modeling purposes. As shown in Supplementary Fig. 3, the residual graph convolution model is constructed based on the graph convolution model, but a layer of residual links is added to each layer. These residual links add the input and output of each layer as the next layer (Eq. 12) because the transmission of information and gradients provides an additional connection channel, which can resolve the smoothness problem. The chosen graph convolution model is a graph convolution model of order *k* based on Chebyshev polynomials^35^. *K* determines the scope of the graph convolution. As shown in Supplementary Fig. 4, when *K* = 0, *T*_0_(***L***) = ***I***, and this model represents only the node itself. When *K* > 0, the model can extract the first-order to *K*th-order neighborhood relations of the predicted grid. For example, when *K* = 2, the model can extract the first-order and second-order neighborhood relations of the predicted grid as follows:

9 $${\varvec{X}}_{l + 1} = relu\left( {\mathop \sum \limits_{k = 0}^{K} \alpha_{k} T_{k} \left( {{\varvec{L}}^{Fu} } \right){\varvec{X}}_{l} } \right)$$

10 $$\left\{ {\begin{array}{*{20}c} {T_{0} \left( {\varvec{L}} \right) = I} \\ {T_{1} \left( {\varvec{L}} \right) = {\varvec{L}}^{Fu} } \\ {T_{2} \left( {\varvec{L}} \right) = 2T_{1} \left( {{\varvec{L}}^{Fu} } \right) - T_{0} \left( {{\varvec{L}}^{Fu} } \right)} \\ \cdots \\ {T_{k} \left( {\varvec{L}} \right) = 2T_{k - 1} \left( {{\varvec{L}}^{Fu} } \right) - T_{k - 2} \left( {{\varvec{L}}^{Fu} } \right)} \\ \end{array} } \right.$$

11 $$relu\left( x \right) = max\left( {0,x} \right)$$

12 $${\varvec{X}}_{l + 1}^{^{\prime}} = {\varvec{X}}_{l + 1} + {\varvec{X}}_{l}$$

where ***X***_*l*_ is the input feature of layer *l* of *N* grids. ***X***_*l*+1_ is the output feature of layer *l* of *N* grids. ${\varvec{X}}_{l + 1}^{{\prime }}$ is the input feature of the *l* + 1 layer of the *N* grids. M is the number of features. *T*_*k*_(·) is a Chebyshev polynomial (Eq. 10). *α*_*k*_ is a trainable parameter. *relu* is the rectified linear unit activation function (Eq. 11), and *max* is the maximum function.

### Temporal dependence modeling

To determine the influence of historical crowd flow dynamics, we use the LSTM model to extract the temporal characteristics^36^. Instead of using the historical crowd flow as input directly for the traditional LSTM model, we use the result of the residential GCNs in the previous step as the input. An LSTM unit consists of a cell and three gates: an input gate, an output gate, and a forget gate. The cell state saves the crowd flow information in this study. The specific calculation process is as follows:

First, the crowd flow matrix ***X***_*t*_ of each grid at time *t* is input as the origin state of the cell, and the output ***h***_*t*−1_ at time *t* − 1 and the input ***X***_*t*_ at the current time *t* are used to calculate the forget gate ***f***_*t*_. The output of this layer is a value between 0 and 1, which is used to determine the crowd flow information retention degree at time *t* − 1.

13 $${\varvec{f}}_{t} = \sigma \left( {{\varvec{W}}_{{\varvec{f}}} \cdot \left[ {{\varvec{h}}_{t - 1} ,{\varvec{X}}_{t} } \right] + {\varvec{b}}_{{\varvec{f}}} } \right)$$

14 $$\sigma \left( x \right) = \frac{1}{{1 + e^{ - x} }}$$

where ***h***_*t*−1_ represents the output at time *t* − 1, which is obtained through an iterative loop calculation. For details, please refer to Eq. (20) in the last step of this process. ***f***_*t*_ indicates the forget gate function at time *t*. ***W***_***f***_ is the weight matrix of the input layer, and the optimal value is obtained through model training. ***b***_***f***_ is the paranoid item of the input layer, and the optimal value is also obtained through model training. *σ* is the sigmoid function (Eq. 14).

Then, the output ***h***_*t*−1_ at time *t* − 1 and the input ***X***_*t*_ at time *t* are used to calculate the input gate *i*_*t*_. The output of this layer is a value between 0 and 1, which is used to determine the degree of retention of the crowd flow at time *t*. In addition, the output ***h***_*t*−1_ at time *t* − 1 and the input ***X***_*t*_ at the current time *t* are used to generate a candidate vector $\tilde{\user2{C}}_{t}$ as follows:

15 $${\varvec{i}}_{t} = \sigma \left( {{\varvec{W}}_{{\varvec{i}}} \cdot \left[ {{\varvec{h}}_{t - 1} ,{\varvec{X}}_{t} } \right] + {\varvec{b}}_{{\varvec{i}}} } \right)$$

16 $$\tilde{\user2{C}}_{t} = tanh\left( {{\varvec{W}}_{{\varvec{C}}} \cdot \left[ {{\varvec{h}}_{t - 1} ,{\varvec{X}}_{t} } \right] + {\varvec{b}}_{{\varvec{c}}} } \right)$$

17 $$\tanh \left( x \right) = \frac{{e^{x} - e^{ - x} }}{{e^{x} + e^{ - x} }}$$

where ***W***_***i***_ and ***W***_***C***_ represent the weight matrices in the input and state update layers, respectively, ***b***_***i***_ and ***b***_***c***_ are corresponding paranoid terms, and the optimal values are obtained through model training. *tanh* is the activation function (Eq. 17).

Then, the cell state is updated: ***C***_*t*−1_ is updated to ***C***_*t*_. The value ***f***_*t*_ of the forget gate is multiplied by the old cell state *C*_*t*−1_, which stores historical crowd flow information, and part of the historical crowd flow information is forgotten. Then, the input gate value ***i***_*t*_ is multiplied by the candidate vector $\tilde{\user2{C}}_{t}$ to store part of the crowd flow information at the current moment. Finally, the two results are added together to determine the new cell state as follows:

18 $${\varvec{C}}_{t} = {\varvec{f}}_{t} *{\varvec{C}}_{t - 1} + {\varvec{i}}_{t} *\tilde{\user2{C}}_{t}$$

Last, the output gate *o*_*t*_ is calculated using the output ***h***_*t*−1_ at time *t* − 1 and the input ***X***_*t*_ at time *t*. The output of this layer is a value between 0 and 1, which is used to determine which parts of the new cell state will be output. Then, the *tanh* function is used to process the cell state ***C***_*t*_, and the processed value is multiplied by the output gate value *o*_*t*_ to obtain the output value as follows:

19 $${\varvec{o}}_{t} = \sigma \left( {{\varvec{W}}_{{\varvec{o}}} \cdot \left[ {h_{t - 1} ,{\varvec{X}}_{{\varvec{t}}} } \right] + {\varvec{b}}_{{\varvec{o}}} } \right)$$

20 $${\varvec{h}}_{t} = {\varvec{o}}_{t} {\text{*tanh}}\left( {{\varvec{C}}_{t} } \right)$$

where ***W***_***o***_ and ***b***_***o***_ are the weight matrix and the paranoid term of the input layer, respectively, and the optimal value is obtained through model training.

### Evaluation and validation

We use the mean absolute error (MAE) and root mean square error (RMSE) and mean absolute percentage error (MAPE) to evaluate the outcomes of the proposed model, as well as other comparative models. MAE refers to the mean value of the absolute error between the predicted value and the actual observed value (Eq. 21). RMSE is the square root of the mean square difference between the predicted value and the actual observed value (Eq. 22). MAPE is a measure of the percentage error of the forecast in relation to the actual observed values (Eq. 23).

21 $$MAE^{i} = \frac{{\mathop \sum \nolimits_{t = 1}^{Q} \left| {y_{t}^{i{\prime}} - y_{t}^{i} } \right|}}{Q}$$

22 $$RMSE^{i} = \sqrt {\frac{{\mathop \sum \nolimits_{t = 1}^{Q} \left( {y_{t}^{i{\prime}} - y_{t}^{i} } \right)^{2} }}{Q}}$$

23 $$MAPE^{i} = \frac{{\mathop \sum \nolimits_{t = 1}^{Q} \frac{{\left| {y_{t}^{i{\prime}} - y_{t}^{i} } \right|}}{{y_{t}^{i} }}}}{Q}$$

where $y_{t}^{i}$ and $y_{t}^{{i{\prime }}}$ represent the observed and predicted crowd flows of grid *i* at time *t*, respectively. *Q* is the predicted sample.

To better deal with the skewness problem (e.g., the Simpson paradox^37^), we added the weighted mean absolute error (WMAE) and the weighted mean absolute percentage error (WMAPE) to evaluate the results.

24 $$WMAE = \mathop \sum \limits_{i = 1}^{N} \left| {y_{t}^{i{\prime}} - y_{t}^{i} } \right|$$

25 $$WMAPE = \frac{WMAE}{{\mathop \sum \nolimits_{i = 1}^{N} \left| {y_{t}^{i} } \right|}}$$

## Experiments and results

### Data and data preprocessing

A mobile phone location dataset of Xining, a city in western China, is adopted in this study. The dataset contains more than 170 million records collected from approximately 0.3 million mobile phone users. This dataset is generated by the incoming or initiated actions of the user and contains a variety of record types (e.g., calls, SMS, internet access, etc.). Each record includes a corresponding timestamp, location and anonymized user ID. The users account for 22% of the population of the city. The dataset covers 4 consecutive work days in August 2018. Each user has 140 records a day on average, which indicates an average temporal interval shorter than 15 min. We extract 2559 base stations from the dataset (Supplementary Fig. 5), of which approximately 96% cover less than 500 m (Supplementary Fig. 6).

The POI data are applied to describe the region function and measure the function similarity between regions, which is ***A***^***P***^ in Section "Graph construction". The data are collected based on the application programming interface (API) of Amap (www.amap.com), which is one of the most popular online map service products in China (Supplementary Fig. 7(a)). The dataset contains 21 categories, such as companies and scenic spots. (Supplementary Table 1).

A road network is applied to measure the connectivity strength between every two regions and construct a spatial connectivity matrix ***A***^***c***^ in Section "Graph construction". The road network data (Supplementary Fig. 7(b)) are downloaded from the OpenStreetMap (www.openstreetmap.com). To reduce the impacts from unevenly distributed small roads, only the expressway, the main road and the secondary road are retained.

To build and examine the proposed model, we first calculate the crowd flow of each grid. To avoid the potential impacts caused by the substantial crowd flow difference between the day and the night derived from the origin dataset, we need to estimate the location of each user at the target timestamps.

First, we divide a day into several fixed time windows and estimate the most likely location of each user in every time window. The mobile phone location records are distributed unevenly in the temporal dimension. In particular, a time window for a user trajectory may contain no record or may have more than one record. To better estimate the most likely location, the record closest to the middle timestamp is retained by referring to the method in Zhao et al.^38^.

Second, we divide the study area into grids and calculate the crowd flow of each grid. The grids are defined based on the left-lower corner location and the fixed size. Each grid is coded by corresponding row and column numbers. For every time window, we calculate the crowd flow by counting the user number for each grid based on its colocation relation.

For the POIs in each grid, the proportions of the 21 categories are calculated. We use the Kolmogorov–Smirnov test (K-S test) to investigate the fitness of the normal distribution and the POI distribution for the grid. The p-value is 0.037, which indicates a normal distribution at the 0.1 significance level but not at the 0.05 or smaller significance level. We further test the distribution by category for each grid and find that 79% of the grid exhibits a normal distribution at the 0.05 significance level. The above results imply that the POI distribution satisfies a normal distribution, and the Pearson correlation coefficient can reasonably describe the similarity of the POIs between grids in general. Therefore, we set the similarity algorithm in Eq. (3) to the Pearson correlation coefficient.

26 $$sim\left( {{\varvec{poi}}^{{\varvec{i}}} ,{\varvec{poi}}^{{\varvec{j}}} } \right) = \left\{ \begin{array}{ll} r_{ij} ,&\quad r_{ij} > 0 \\ 0, &\quad r_{ij} \le 0 \\ \end{array} \right.$$

27 $$r_{ij} = \frac{{\mathop \sum \nolimits_{z = 1}^{Z} \left( {poi_{z}^{i} - \overline{{{\varvec{poi}}^{{\varvec{i}}} }} } \right)(\left( {poi_{z}^{j} - \overline{{{\varvec{poi}}^{{\varvec{j}}} }} } \right)}}{{\sqrt {\mathop \sum \nolimits_{z = 1}^{Z} \left( {poi_{z}^{i} - \overline{{poi^{i} }} } \right)^{2} } \sqrt {\mathop \sum \nolimits_{z = 1}^{Z} \left( {poi_{z}^{j} - \overline{{{\varvec{poi}}^{{\varvec{j}}} }} } \right)^{2} } }} \in \left[ { - 1,1} \right]]$$

where $poi_{z}^{i}$ and $poi_{z}^{j}$ represent the ratio of POI type *z* in grid *i* and grid *j*, respectively; ${\text{z}} \in \left[ {1,{\text{Z}}} \right]; {\text{Z}}$ represents the type number of all POIs. ${\varvec{A}}_{ij}^{P}$ represents the functional similarity between *v*^*j*^ and *v*^*j*^. As we said before, grids with the same function will improve the accuracy of the prediction grid.

Referring to existing research^39^, we set the negative *r* value as zero.

We use the network analyst module of ArcGIS 10.2 to organize the road network and calculate the shortest path between the grids. Then, the connectivity strength matrix can be built by Eqs. (4) and (5).

### Parameter settings

In the experiment, we choose the data from the first three days as the training set and the data from the last day as the test set. The grid size, the time window and the connectivity threshold are three basic parameters that need to be set. First, we choose 500 m to define the grid size by jointly considering the spatial resolution of the dataset and the commonly used size in previous studies^40,41^. Second, considering the temporal intervals of the mobile phone dataset, we set the most fine time window as 15 min and further analyzed and compared the outcomes for 30 min, 45 min, and 60 min. Third, the distance threshold δ in the connectivity graph is set based on the daily movement speed (i.e., 80 km/h in this study) in urban areas and the predicted time window width.

The parameters of the proposed FPM-geo model mainly include the learning rate, batch processing volume, number of training iterations, number of layers and graph convolution order. In this experiment, the learning rate is set to 0.001, the batch size is set to 16, the number of training iterations is set to 2000, the number of graph convolution layers is set to 3, and the graph convolution order is set to 2 (Supplementary Fig. 8).

### Prediction results

**Figure 1:**
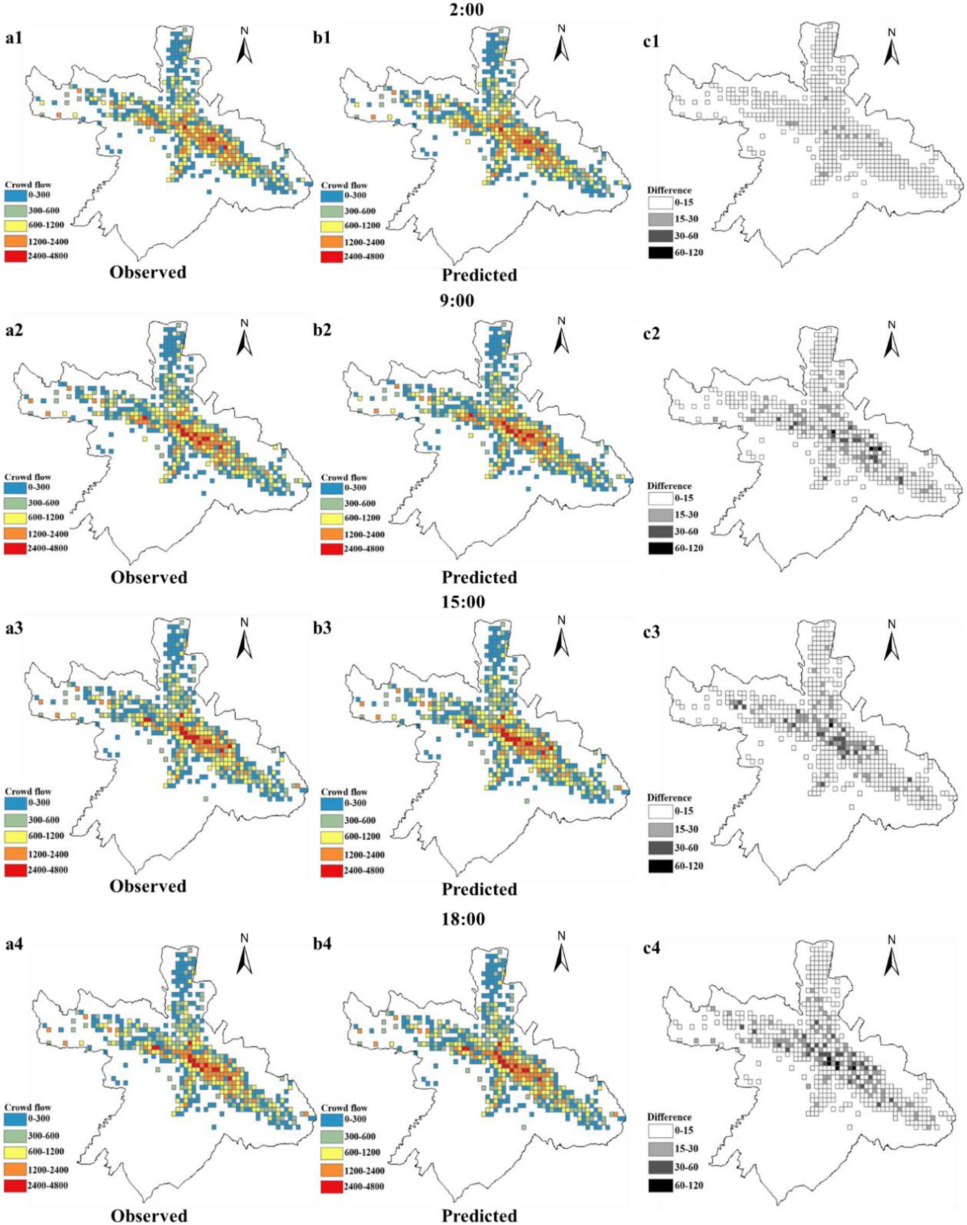
Crowd flow prediction results.

Figure 1 shows the results of the urban crowd flow prediction with a 500-m grid size and a 15-min prediction step. The urban crowd flow is concentrated mainly along the two cross rivers in the downtown area. The absolute prediction differences during the night tend to be smaller than those during the daytime. Moreover, the grids with large absolute prediction differences are distributed more sparsely during the morning and evening rush hours and concentrate on the center area during the working hours.

### Comparative analysis

To better reflect the prediction performance, 4 prediction time steps are applied, namely, 15 min, 30 min, 45 min, and 60 min. We compare the proposed model (FPM-geo) with the following 5 prediction methods: SVR is a multivariate extension of the autoregressive model, which is capable of modeling correlations between regions. LSTM is a variant of traditional RNN. It can effectively capture the semantic association between long sequences and alleviate the phenomenon of gradient disappearance or explosion compared with classical RNN. In GCN, features are extracted from graph data and used to make predictions. TGCN and STMGCN are both graph convolution based traffic prediction models. TGCN only considers a single spatial relationship. STMGCN considers multiple spatial relationships.

The result indicates that the proposed FPM-geo outperforms all 5 selected models for each prediction time step (Table 1). Among these models, machine learning models (i.e., SVR) have the lowest prediction accuracy. Compared with the model that only considers temporal features (i.e., LSTM) or the model that only considers spatial features (i.e., GCN), the models that integrate both spatial and temporal features (i.e., TGCN, STMGCN) have higher prediction accuracy. For the model that only considers limited spatial and temporal features (i.e., STMGCN), our model achieves a better prediction performance by integrating multiple geographical features. For example, the FPM-geo outperforms the STMGCN and LSTM with decreased RMSE values of 4.67% and 23.12%, respectively, with a prediction time step of 15 min.

**Table 1:** Performance comparison among different prediction models.

T (min)	Metric	SVR	LSTM	GCN	TGCN	STMGCN	FPM-geo
15	MAE	12.56	9.46	8.93	8.72	8.25	7.90
	RMSE	20.86	16.18	14.89	14.70	13.05	12.44
	MAPE	0.0431	0.0338	0.0333	0.0327	0.0326	0.0304
	WMAE	20.87	15.89	15.02	14.52	13.42	12.53
	WMAPE	0.0170	0.0130	0.0122	0.0118	0.0109	0.0101
30	MAE	19.26	14.42	14.06	12.99	12.26	12.18
	RMSE	33.51	25.83	24.62	22.82	20.81	19.93
	MAPE	0.0645	0.0492	0.0450	0.0439	0.0431	0.0427
	WMAE	42.99	25.67	24.84	23.09	20.56	19.97
	WMAPE	0.0352	0.0210	0.0203	0.0188	0.0167	0.0162
45	MAE	25.67	18.99	18.25	17.26	16.24	15.45
	RMSE	47.44	35.11	32.29	30.53	27.82	26.76
	MAPE	0.0780	0.0545	0.0527	0.0526	0.0515	0.0502
	WMAE	56.58	33.67	31.77	30.69	27.97	25.82
	WMAPE	0.0463	0.0275	0.0259	0.0250	0.0228	0.0210
60	MAE	30.22	23.55	22.92	21.50	20.5	19.31
	RMSE	60.47	43.88	40.30	39.78	36.13	34.95
	MAPE	0.0909	0.0684	0.0635	0.0611	0.0599	0.0588
	WMAE	69.56	42.67	41.21	40.10	37.79	33.15
	WMAPE	0.0569	0.0348	0.0337	0.0327	0.0309	0.0270

The proposed FPM-geo model exhibits stronger robustness in MAE, RMSE, MAPE, WMAE and WMAPE than the other models by the changes in the prediction steps. The MAE, RMSE, MAPE, WMAE and WMAPE values of each model increase with the prediction time window. However, the prediction errors of the FPM-geo model are lower than those of the other models. In particular, when the prediction step lengths are 45 min and 60 min, the MAE, RMSE, MAPE, WMAE and WMAPE values of the FPM-geo model are considerably lower than those of the other models.

### The effects of spatial relationships

The three typical geographic characteristics make the main contribution to the effectiveness of the proposed model, especially for areas or time periods with complex population flows. Each geographic characteristic contributes varying effectiveness for different places. To further investigate the effectiveness of the three geographic characteristics, we compare the prediction performance of the pure LSTM (no geographic characteristic is considered), the original FPM-geo (all three geographic characteristics are integrated) and the modified FPM-geo by removing different geographic characteristics. The FPM-geo-J, FPM-geo-F, and FPM-geo-C in Table 2 indicate the modified models in which the proximity relationship (J), the functional similarity relationship (F) and the road connectivity relationship (C) are removed, respectively. Table 2 indicates that the LSTM model prediction errors are the largest, and each modified model has smaller errors than the LSTM but larger errors than the FPM-geo model. The results imply that each spatial relationship contributes a positive effect on the prediction performance.

**Table 2:** Influence of spatial relationships on prediction.

Method	MAE	RMSE	MAPE
LSTM	9.46	16.18	0.0334
FPM-geo-J	8.37	13.42	0.0326
FPM-geo-F	8.20	12.79	0.0324
FPM-geo-C	8.17	13.01	0.0324
FPM-geo	7.9	12.44	0.0304

In this table, FPM-geo-J is the model without proximity. FPM-geo-F is the model without functional similarity. FPM-geo-C is the model without road network connectivity.

**Figure 2:**
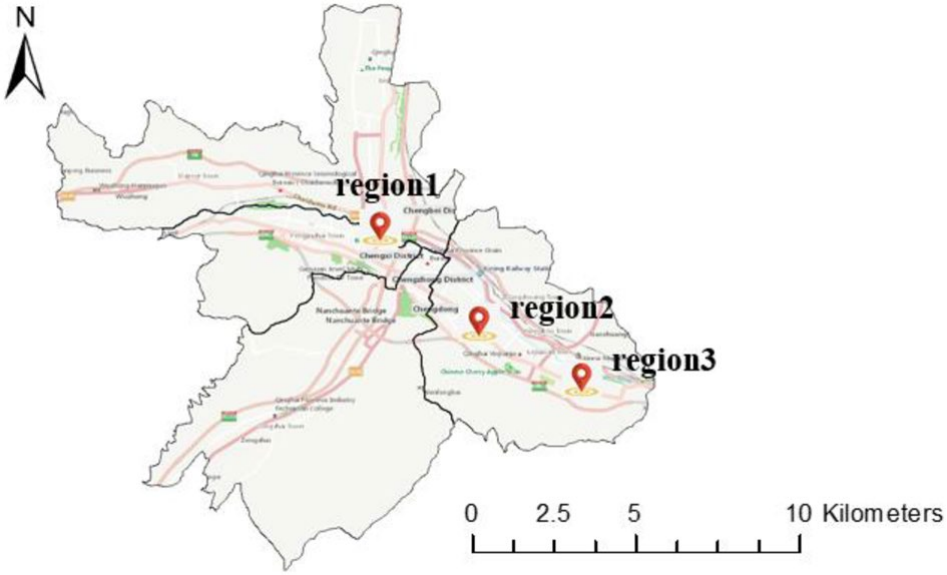
Three typical regions.

**Figure 3:**
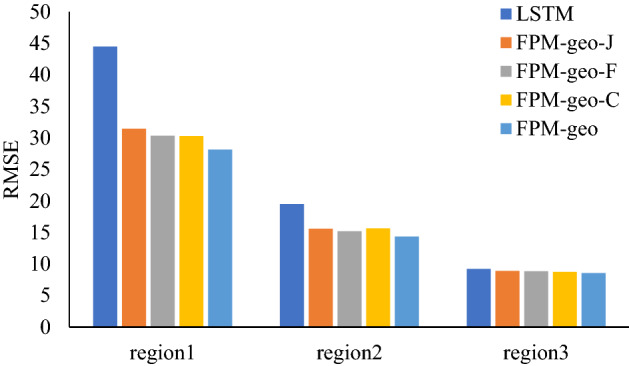
RMSEs of typical regions with different considered spatial relationships.

**Figure 4:**
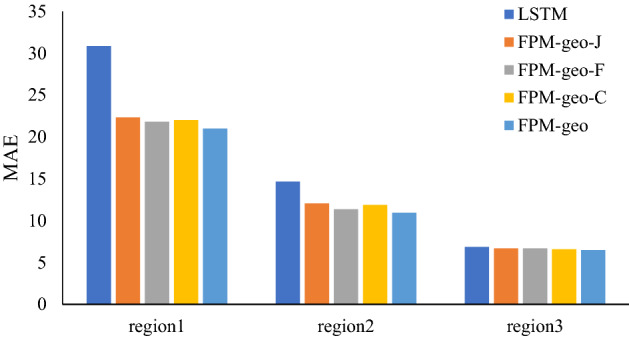
MAEs of typical regions with different considered spatial relationships.

**Figure 5:**
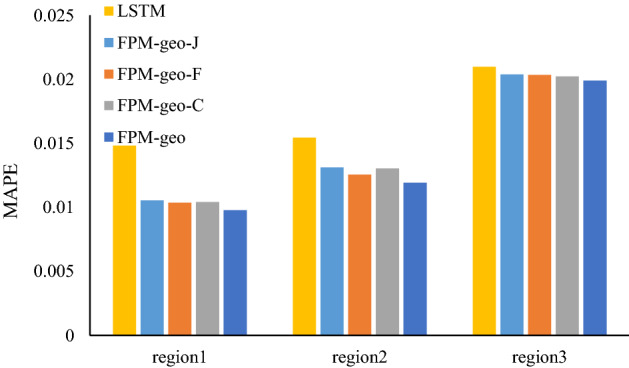
MAPEs of typical regions with different considered spatial relationships.

To further compare the contribution of each geographic characteristic to crowd flow prediction for different places, we select three typical regions, i.e., the city center (Region 1), suburbs (Region 2) and outer suburbs (Region 3), for further analysis (Fig. 2). As shown in Figs. 3, 4 and 5, for the city center, removing the proximity relationship (FPM-geo-J) results in less improvement to the LSTM than that for the other two modified models. This indicates that the proximity relationship plays a more critical role in the crowd flow prediction model in the urban center area (Region 1). Similarly, the connectivity and proximity of the road network make greater contributions in suburban areas (Region 2). For the outer suburbs, the overall crowd flow is small, and there are very few people moving, so the three geographic characteristics have relatively small impacts.

### The spatial distribution of the prediction error

**Figure 6:**
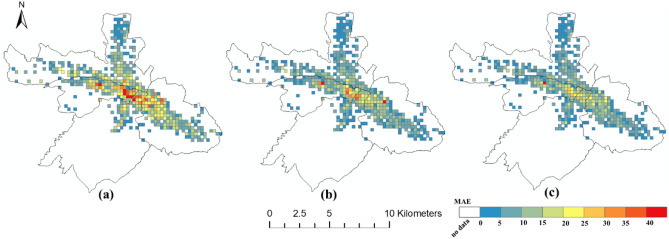
Spatial distributions of the MAEs of different models: (**a**) SVR; (**b**) TGCN; (**c**) FPM-geo.

**Figure 7:**
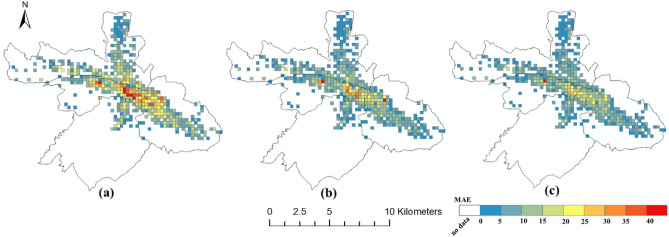
Spatial distributions of the RMSEs of different models: (**a**) SVR; (**b**) TGCN; (**c**) FPM-geo.

**Figure 8:**
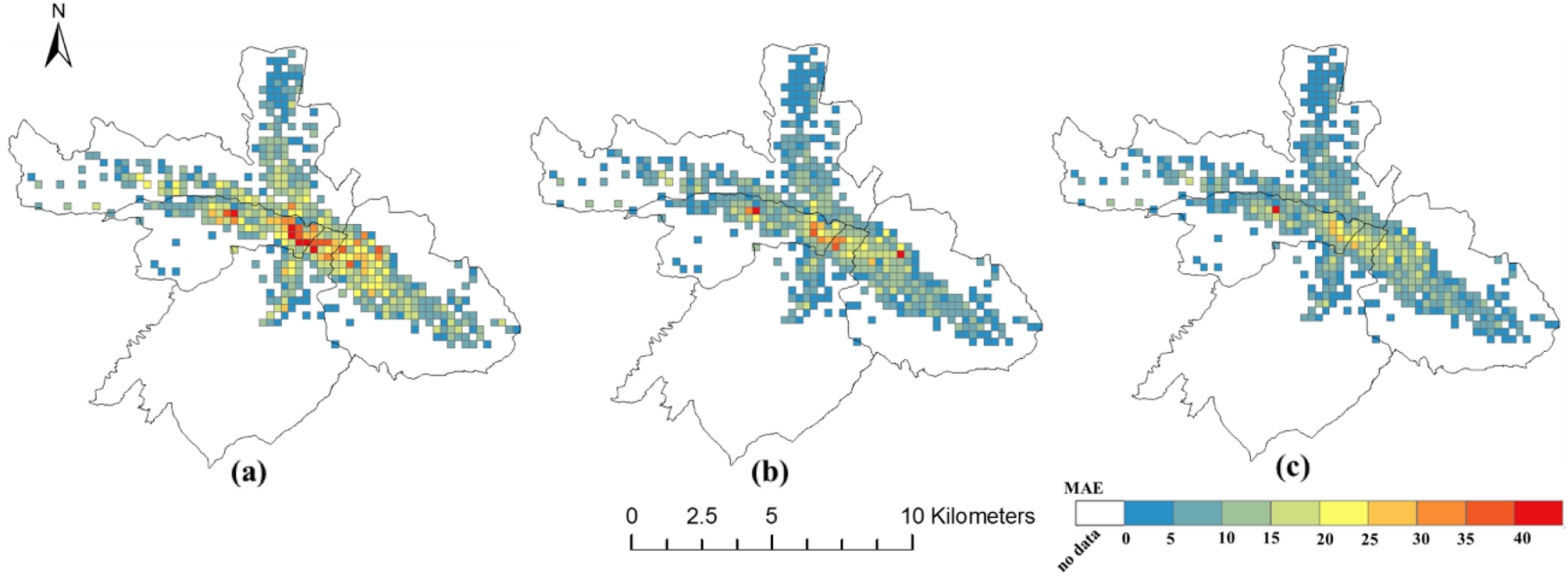
Spatial distributions of the MAPEs of different models: (**a**) SVR; (**b**) TGCN; (**c**) FPM-geo.

**Figure 9:**
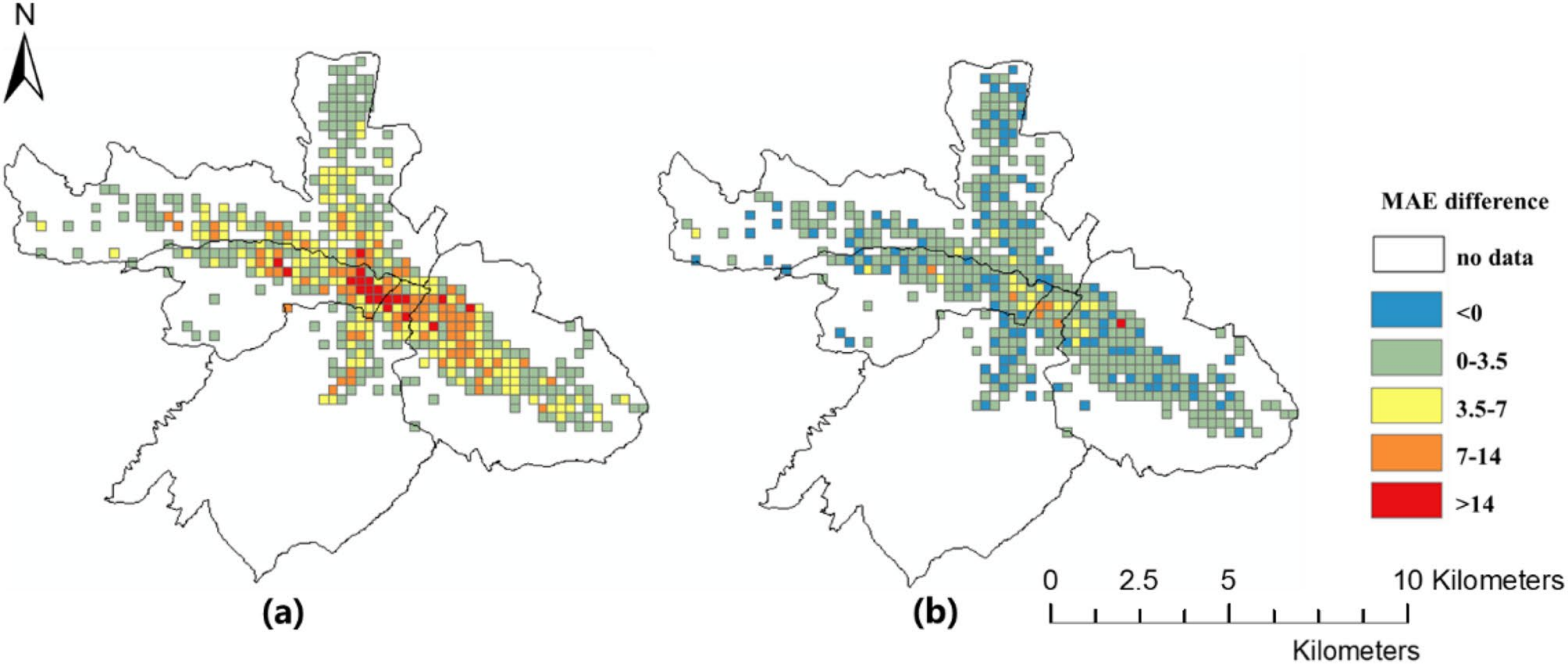
Spatial distributions of the MAE differences among different models: (**a**) FPM-geo and SVR; (**b**) FPM-geo and TGCN.

**Figure 10:**
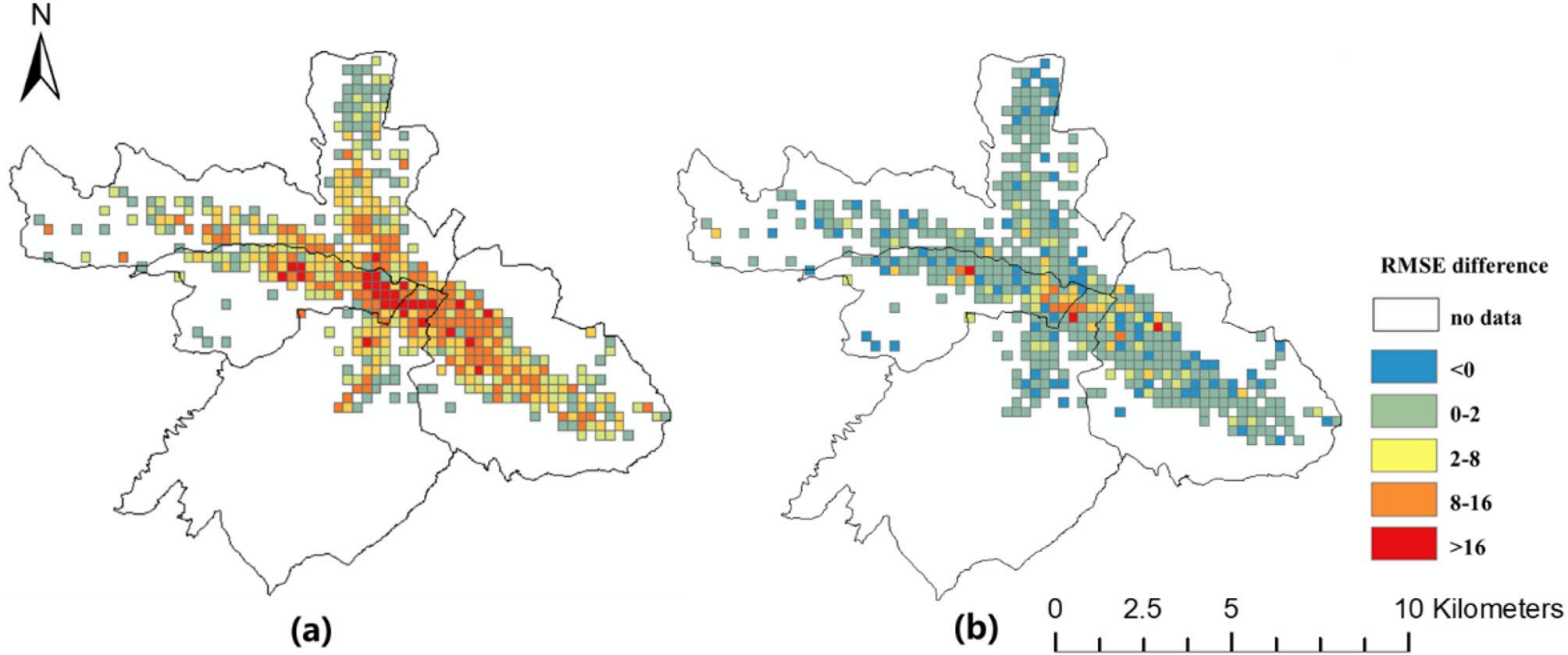
Spatial distributions of the RMSE differences among different models: (**a**) FPM-geo and SVR; (**b**) FPM-geo and TGCN.

**Figure 11:**
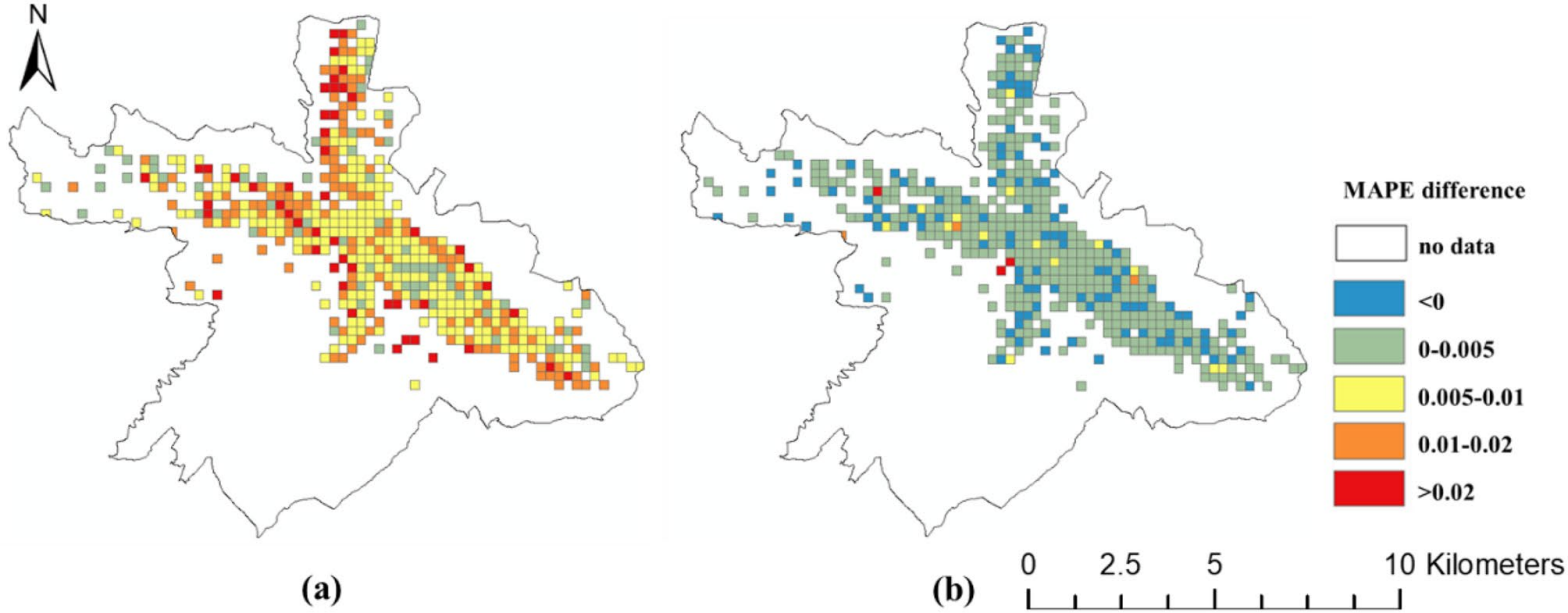
Spatial distributions of the MAPE differences among different models: (**a**) FPM-geo and SVR; (**b**) FPM-geo and TGCN.

The spatial distributions of the prediction errors are shown in Figs. 6, 7 and 8. The errors of the proposed FPM-geo are lower than those of the other two methods for most grids (Figs. 9, 10 and 11). For the downtown areas with high crowd flow density located at the center of the city, the improvement is more obvious. In terms of the spatial distribution, the grids with high RMSE and MAE are mainly concentrated in the central areas and the western part of the city (Figs. 6 and 7). These areas share a common characteristic of high crowd flow density. Residential, office and commercial buildings concentrate in limited space and mix with each other. The crowd flow flows vary in travel purpose (e.g., commuting travel and entertainment travel), as well as travel distance (e.g., short travel from local citizens and long travel from suburban citizens). Therefore, predicting the crowd flow in these areas needs to consider the complex nature of the flow. This is the main reason why the proposed FPM-geo outperforms the other methods in this study, especially for downtown areas. For the grids with low crowd flow density located in suburban areas, the crowd flow is relatively simple. Both the proposed method and the comparative methods share a low and similar performance. In contrast to RMSE and MAE, grids with higher MAPE are mostly distributed in suburbs with less crowd flow (Fig. 8). This indicates that high prediction errors mainly occur in grids with small crowd flows. The risk of emergency urban events is more prevalent in densely populated areas. The MAPEs of several models are smaller on grids with high crowd flow, indicating the effectiveness of several models in practical applications. In areas with large crowd flows, FPM-geo also outperforms the other methods, which further reflects its performance.

**Figure 12:**
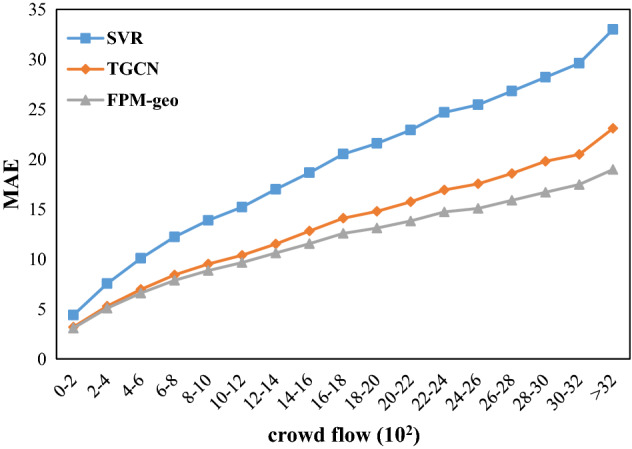
MAE distributions based on the number of people in the grid.

**Figure 13:**
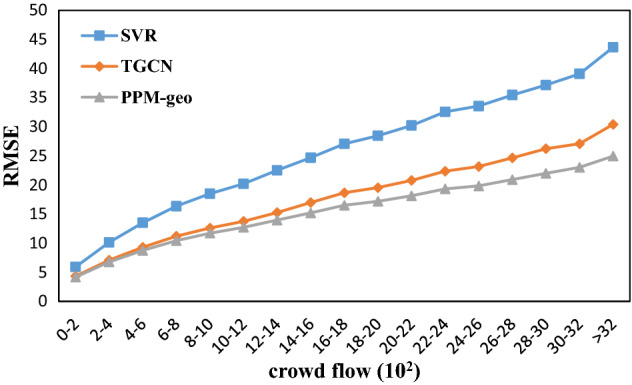
RMSE distributions based on the number of people in the grid.

The spatial distribution of the MAE and RMSE implies a positive relation between the prediction errors and the crowd flow. Further investigation shows that both the MAE and the RMSE of the three models increase with the crowd flow (Figs. 12 and 13). However, the FPM-geo outperforms the other two methods in both absolute prediction errors and the robustness of the good performance with the increase in crowd flow. For example, when the crowd flow changes from the range [0, 200) to the range [2000, 2200], the RMSE of FPM-geo increases from 4.17 to 18.13, TGCN increases from 4.32 to 20.78, and SVR increases from 5.91 to 30.21. The nearly 2000 increase in crowd flow leads to 13.96, 16.46 and 24.70 decreases for the FPM-geo, TGCN and SVR models, respectively. The FPM-geo exhibits 84.81% and 56.52% of the prediction performance loss caused by the same crowd flow increase for the TGCN and SVR models, respectively. When the crowd flow reaches 2000, the RMSE of the FPM-geo is only 87.25% and 60.01% of those for the TGCN and SVR models, respectively.

**Figure 14:**
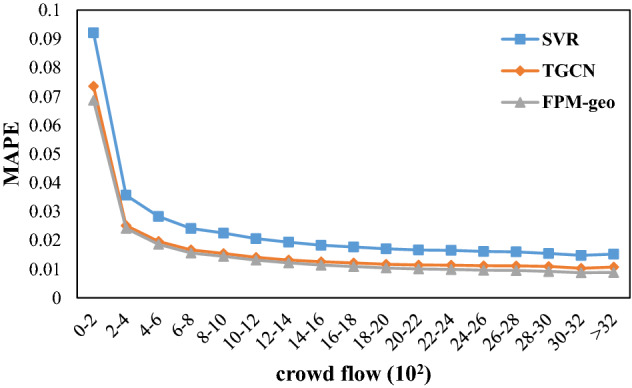
MAPE distributions based on the number of people in the grid.

The spatial distribution of MAPE implies a negative correlation between prediction error and crowd flow. Further investigation showed that the MAPE of all three models decreased with increasing crowd flow (Fig. 14). However, FPM-geo outperforms the other two methods in both absolute prediction error and robustness of good performance with decreasing crowd flow. For example, when the crowd flow changes from the [3000, 3200] range to the [0, 200] range, the MAPE of FPM-geo increases from 0.009 to 0.069, TGCN increases from 0.01 to 0.074, and SVR increases from 0.015 to 0.092. The reduction in crowd flow of nearly 3000 resulted in decreases of 0.06, 0.063 and 0.077 for the FPM-geo, TGCN and SVR models, respectively. FPM-geo exhibits prediction performance losses of 94.69% and 77.46% due to the same crowd flow reduction for the TGCN and SVR models. When the crowd flow is lower than 200, the RMSE of PPMFPM-geo is only 93.35% and 74.54% of the TGCN and SVR models, respectively.

### Time distribution of the prediction error

**Figure 15:**
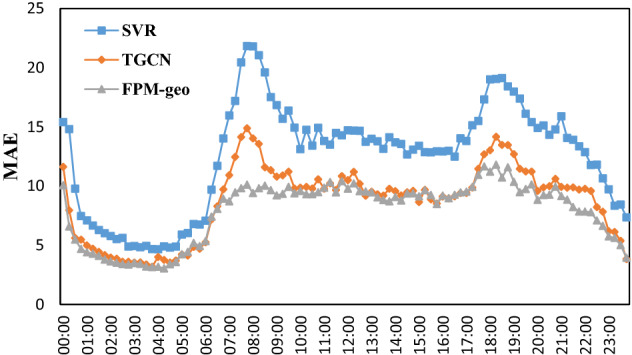
Time distribution of the MAE of each model.

**Figure 16:**
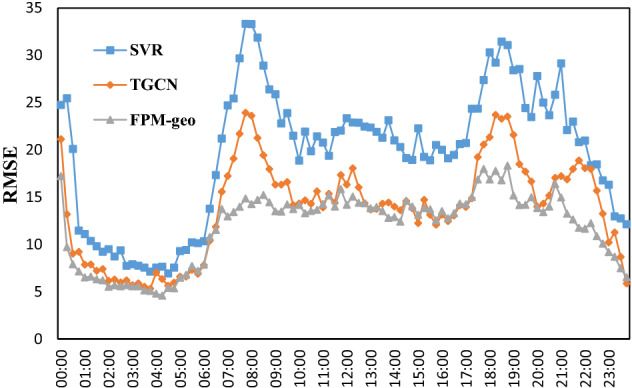
Time distribution of the RMSE of each model.

**Figure 17:**
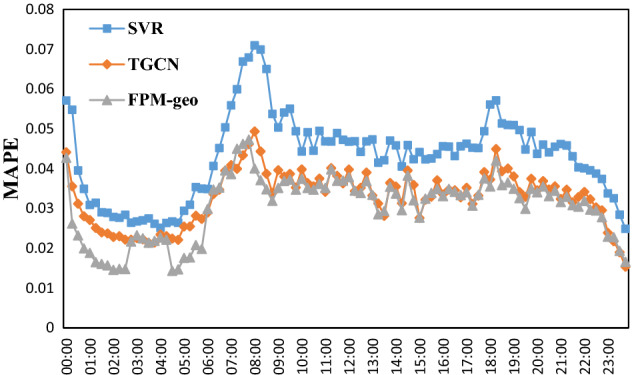
Time distribution of the MAPE of each model.

**Figure 18:**
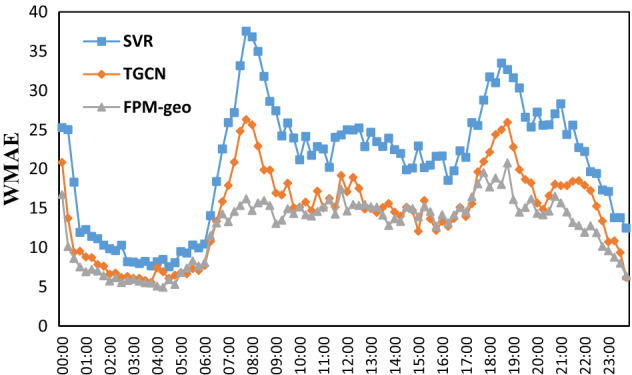
Time distribution of the WMAE of each model.

**Figure 19:**
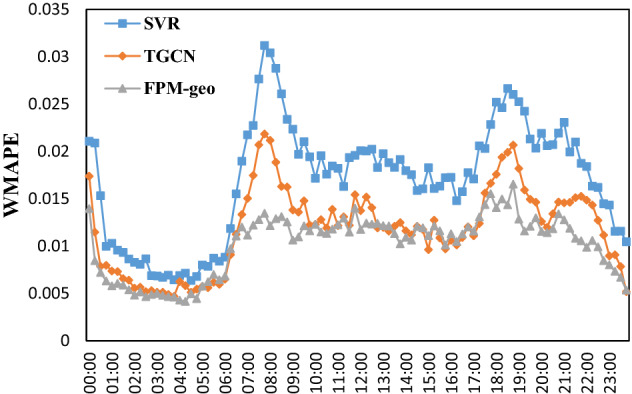
Time distribution of the WMAPE of each model.

The time distributions of the two prediction errors are shown in Figs. 15, 16, 17, 18 and 19. The RMSE, MAE, MAPE, WMAE, and WMAPE of the TGCN and SVR models exhibit three peaks in the morning rush hours (7:00–9:00), evening rush hours (17:00–19:00) and the fortnight (21:00–22:00). The proposed FPM-geo has an obviously lower RMSE and only one small peak during 18:00–19:00, which indicates its overall effectiveness in crowd flow prediction. Specifically, during the morning peak, the RMSE of the FPM-geo remains below 15, which is over 22% lower than that of TGCN and over 47% lower than that of SVR. In the evening peak, the RMSE of the FPM-geo remains below 19, which is over 12% lower than that of the TGCN and over 34% lower than that of SVR.

In general, crowd flow prediction during the daytime exhibits higher error than that during the night. We believe that the underlying reason points to the diversity of travel during the daytime. For example, during the morning and evening rush hours, commuting travel contributes the most to the crowd flow. Citizens from suburban areas travel to downtown areas. The impacts of the crowd flow from distant areas increase considerably. The FPM-geo can better address the spatial and temporal features than the SVR and TGCN and outperforms the other two models in crowd flow prediction. During the night, especially from 1:00 to 6:00, the travel volume remained at a very low level. The impacts from other regions are small, and the improvement of the proposed FPM-geo is also not strong.

## Conclusion and discussion

In this paper, we propose an urban crowd flow spatial distribution prediction model by integrating multiple geographic characteristics. The residual multigraph convolution network is applied to integrate the proximity, functional similarity and connectivity relationships that affect the crowd flow prediction effectiveness. LSTM is applied to model the temporal features of the local crowd flow dynamics. Four days of data with more than 0.3 million users validated the effectiveness of the proposed FPM-geo by comparative analysis with six typical methods in existing studies. The model proposed in this paper integrates three geographic features to improve the performance of urban crowd flow prediction. We analyze the impact of several geographical features on the prediction accuracy from different perspectives and provide some new insights for related research on urban crowd flow prediction.

First, the proposed method can make a contribution to applications based on the prediction of crowd flows due to its performance in crowd flow prediction in both the temporal dimension and the spatial dimension. In fact, rapid changes in crowd flow in space and time can create problems in public safety, response to extreme climate events, epidemic control, and traffic management, etc. For example, stampedes occurred during the Hajj pilgrimage to Mecca in Saudi Arabia in 2015 and during the Shanghai Bund. Recently, stampedes occurred in Seoul, South Korea. If the crowd flow can be predicted in advance, protection policies could be implemented earlier. We can prevent such catastrophic events from happening or reduce their probability. The prediction of the crowd flow can also contribute to higher urban transportation efficiency. The travel demand to support the vehicle scheduling optimization operation is usually derived from the history travel log. Due to multiple travel modes or various travel service companies, related data can hardly capture the overall travel demand appropriately. The estimated travel demand will reach the upper bound limited by the current service supplies. However, the crowd prediction method can provide a better estimation for the travel demand because it reflects the amount of users.

Second, the effectiveness of the three factors and the spatial and temporal patterns of the prediction performance have high potential generalization in other areas with similar spatial distribution patterns in terms of road networks and urban functions. In general, Xining exhibits “single center” patterns in its spatial distribution of urban functions and road networks. The major government departments, the best hospitals and the most popular shopping malls are all concentrated around a cross area of “two developed axes” along rivers across the city. The road network density and the population density are both the highest in the center area and gradually decrease when moving toward the suburbs. The distribution patterns of the above features are common in cities in China (Beijing, Shanghai, Chengdu, etc.), as well as in the world (e.g., the London, Paris, Tokyo). To better reveal the effectiveness of the selected factors, three typical regions (i.e., center area, suburbs and the outer suburbs) have been selected to compare the contribution of different factors. Therefore, we believe that the proposed model can still work and the three spatiotemporal factors can also contribute a positive impact on improving the crowd prediction performance.

There are still some shortcomings in this paper that need further research. First, the effectiveness of the model on weekends or holidays has not been tested due to dataset limitations. Considering the higher diversity of weekend travel patterns, we believe that the proposed model can achieve better performance than the model chosen in this study. Second, due to the data, we only considered three geographical features. If other urban data (such as social life data, health data, etc.) can be collected in the future, we can better understand the relationship between the urban environment, human activities and complex interactions. Third, the potential overfitting issue of the proposed model requires further testing due to the limited time period and spatial extent of the dataset. It will be promising and valuable to test the performance of the proposed model for other cities.

## Supplementary Information

Supplementary Information.

## Data Availability

The data presented in this study are available on request from the corresponding author.
