# Supplementary material for: An urban crowd flow model integrating geographic characteristics
Source: Sci Rep. 2023 Jan 30;13:1695. doi: 10.1038/s41598-023-29000-5 (PMC9886992; doi:10.1038/s41598-023-29000-5)
Supplement: Supplementary file 1 — Supplementary Information. [file 41598_2023_29000_MOESM1_ESM.docx]

**An urban crowd flow model integrating geographic characteristics**

**Authors:** Yu Zhang^1,2,3^, Sheng Wu^1,3,4^, Zhiyuan Zhao^1,3,4,*^,Xiping Yang^5^ and Zhixiang Fang^2^

**Affiliations:**

^1^Academy of Digital China (Fujian), Fuzhou University

^2^State Key Laboratory of Information Engineering in Surveying, Mapping and Remote Sensing, Wuhan University.

^3^Key Laboratory of Spatial Data Mining and Information Sharing

^4^Ministry of Education Fujian Collaborative Innovation Center for Big Data Applications in Governments

^5^School of Geography and Tourism, Shaanxi Normal University

***Corresponding author:** Zhiyuan Zhao

E-mail: [zyzhao@fzu.edu.cn](mailto:zyzhao@fzu.edu.cn)

This file includes:

- Figure S1-S9 (Supplementary figures)

-Table S1 (POI data used in the study)


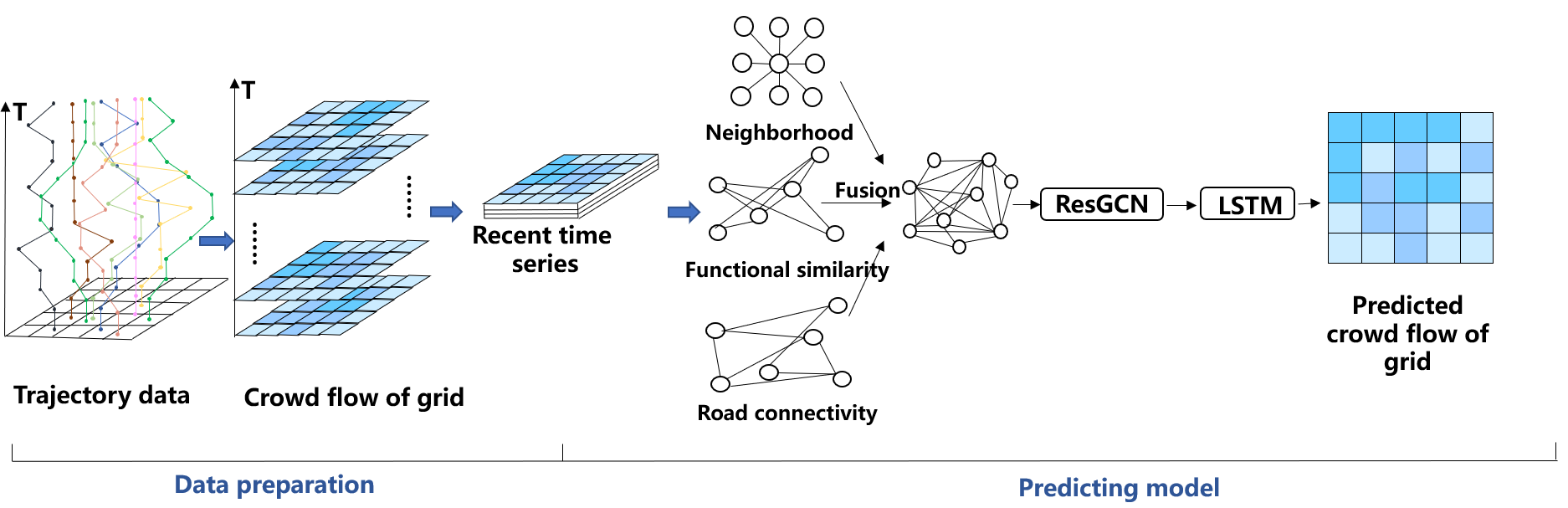


Fig. S1. Framework of the urban crowd flow Prediction Model integrating geographic characteristics (PPM-geo)


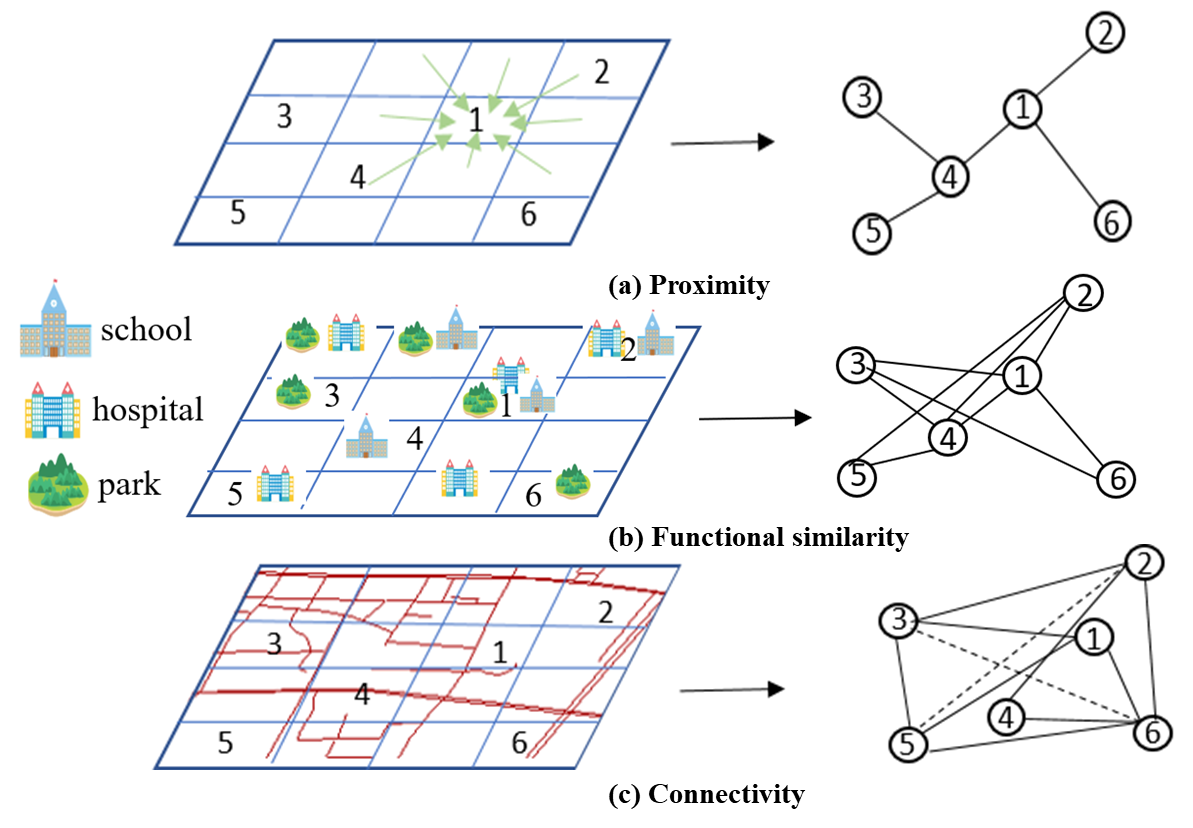


Fig. S2. Different geographic characteristics


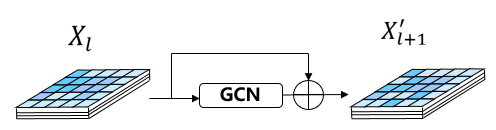


Fig. S3. Residual graph convolution


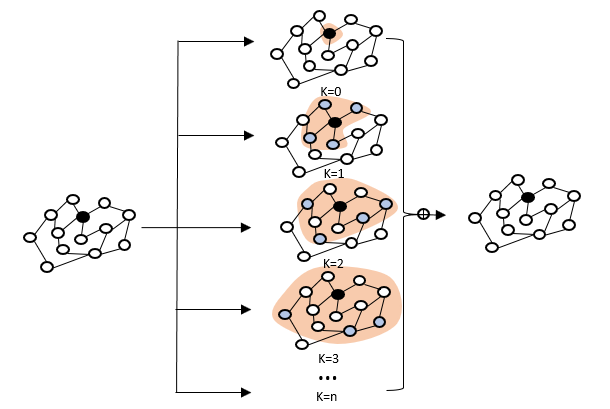


Fig. S4. Graph Convolution


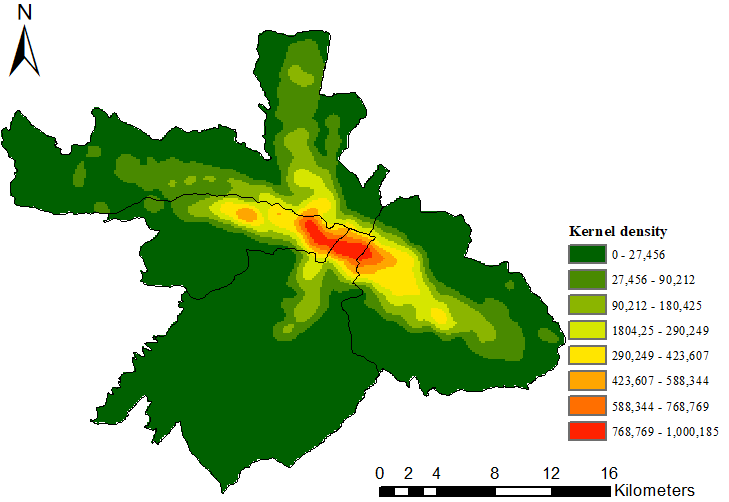


Fig. S5. Kernel density graph of the base station

Fig. S6. Probability distribution of (a) the coverage radius of the base station and (b) the average time intervals between adjacent records

Table S1. POI data example for Xining

| **ID** | **Type** | **Instance** | **Longitude** | **Latitude** |
| --- | --- | --- | --- | --- |
| 1 | Companies | Iron Army Property Company | 101.76***7 | 36.64***7 |
| 2 | Address information | Building 147, Lok Ka Wan Village | 101.87***2 | 36.56***9 |
| 3 | Motorcycle service | Suzuki motor | 101.80***0 | 36.62***3 |
| 4 | Sports leisure service | XBOS Dynamic experience center | 101.75***6 | 36.6xxx4 |
| 5 | Life service | Wangtu Copy shop | 101.77***1 | 36.63***2 |
| 6 | Government agencies and social organizations | Xiaoqiao Street Police Station Police Room | 101.74***0 | 36.64***0 |
| 7 | Scientific, educational and cultural services | Qinghai Artists Association Gallery | 101.78***0 | 36.62***4 |
| 8 | Car service | Che Mei Jia car decoration | 101.79***8 | 36.60***0 |
| 9 | Accommodation services | Xining Jiayuan Hotel | 101.78***1 | 36.62***5 |
| 10 | Shopping services | Zhao Aman's shop | 101.78***7 | 36.62***71 |
| 11 | Car sales | Qinghai Haitong Automobile Trading Co. LTD | 101.74***5 | 36.68***2 |
| 12 | Commercial housing | Jia Hao square | 101.75***9 | 36.62***8 |
| 13 | Healthcare services | Magang Medical Clinic | 101.72***3 | 36.63***8 |
| 14 | Transport Facilities Services | Central Square (North) Station (Bus Station) | 101.77***4 | 36.62***7 |
| 15 | Traffic facilities | Xining Cadres Rest Center (Southwest Gate) | 101.75***4 | 36.62***6 |
| 16 | Food and beverage service | Tengshi steamed bread (Gucheng Tai Head Shop) | 101.76xxx8 | 36.62xxx3 |
| 17 | Vehicle maintenance and repair | Qinghai Jinyubao Automobile Sales and Service Co. LTD | 101.86***8 | 36.56***3 |
| 18 | Scenic spot | Liu Jiazhai Mosque | 101.71***5 | 36.63***9 |
| 19 | Public facilities | Public toilet | 101.80***0 | 36.61***7 |
| 20 | Financial and insurance services | Agricultural Bank of China (Gucheng Tai Branch) | 101.75***3 | 36.62***8 |
| 21 | Indoor facilities | Checkstand | 101.75***6 | 36.63***6 |


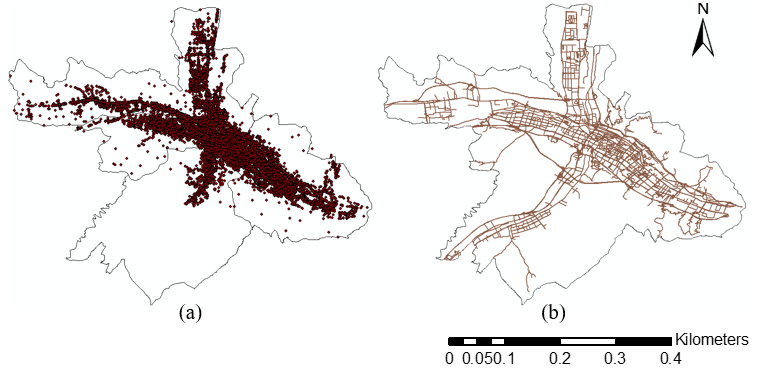


Fig. S7. Distribution of the road network and POI

Fig. S8. The accuracy of different K values
